# Prognostic significance of coagulation factor activity in acute stroke: a retrospective cohort study

**DOI:** 10.3389/fmed.2026.1860924

**Published:** 2026-05-29

**Authors:** Yunyan Ye, Yisha Wang, Huan Wang, Lei Zhou, Shengyi Shi, Xuguang Chen

**Affiliations:** Department of Emergency, Ruijin Hospital, Shanghai Jiao Tong University School of Medicine, Shanghai, China

**Keywords:** coagulation factor, hemostasis, prognosis, stroke, stroke outcome

## Abstract

**Background:**

The coagulation system plays a critical role in the pathophysiology of stroke, extending beyond thrombosis to involve inflammatory immunity and microcirculatory regulation.

**Objective:**

This study aimed to evaluate the association between the activities of coagulation factors VII, IX, XI, and XII and 90-day outcomes in ischemic and hemorrhagic strokes.

**Methods:**

In this retrospective cohort study of 195 stroke patients recruited from May 2019 to March 2023, coagulation factors were measured within 24 h of admission. Multivariable logistic regression analysis was employed to assess their independent associations with post-stroke outcomes.

**Results:**

Out of 195 screened patients, 171 were included in the final analysis. Compared with patients with ischemic stroke (IS), those with hemorrhagic stroke (HS) were younger and presented with higher stroke severity, more severe consciousness disturbance, and higher INR levels (all *p* < 0.05). In the IS group, unfavorable 90-day prognosis was associated with higher levels of FXI and FXII activity (*p* < 0.05), but these associations disappeared after adjustment. In the HS group, unfavorable prognosis was associated with lower FVII and FIX activity, as well as higher FXI and FXII activity (*p* < 0.05). Multivariable logistic regression analysis revealed that in HS patients, after adjusting for potential confounders, FVII (OR = 0.464, 95% CI: 0.250–0.863, *p* = 0.015) and FIX activity (OR = 0.498, 95% CI: 0.249–0.998, *p* = 0.049) remained independently negatively associated with an unfavorable prognosis, while FXII activity (OR = 2.725, 95% CI: 1.098–6.762, *p* = 0.031) was independently positively associated with an unfavorable prognosis. Additionally, FXI activity in HS patients was weakly correlated with GCS and NIHSS scores.

**Conclusion:**

Early coagulation factor activity showed subtype-specific associations with 90-day functional outcome, particularly in hemorrhagic stroke.

## 1 Introduction

As a leading cause of disability and mortality worldwide, the pathophysiology of stroke is closely linked to the activation of the coagulation system (1, 2). In recent years, the role of coagulation factors in post-stroke neural injury and repair has garnered increasing attention. Research indicates that the coagulation system not only participates in thrombus formation but also influences prognosis through pathways such as inflammatory immune regulation. For example, coagulation factor XII (FXII) activates the intrinsic coagulation pathway, promotes pathological thrombosis, and exacerbates microcirculatory impairment in the ischemic penumbra. Its activation product, FXIIa, disrupts the blood–brain barrier and worsens cerebral edema via the kallikrein-kinin pathway (3). In contrast, coagulation factor VII (FVII) may play a protective role in hemorrhagic stroke by forming the TF-FVIIa-FXa complex, which stabilizes microvascular endothelium and reduces hematoma expansion (4). FIX and FXI play key roles in the formation and growth of thrombus, and FXI is a potential therapeutic target (5, 6). However, systematic comparison of the prognostic values of these coagulation factors in different stroke types is currently lacking (7), and there is a deficiency in in-depth analysis regarding the interplay between these coagulation factors and key clinical indices (8). Based on biological plausibility, we screened FVII, FIX, FXI, and FXII to address these gaps by systematically assessing the activity levels of these coagulation factors in different stroke cohorts. This study aimed to bridge these gaps by exploratory evaluation of the relationship between the activity of these coagulation factors and the 90-day clinical prognosis of patients with IS and HS. We seek to: clarify the distinct prognostic value of these specific coagulation factors; identify whether specific coagulation profiles independently predict neurological disability; and explore the mechanisms underlying these associations, thereby providing a theoretical basis for future phenotype-specific therapeutic interventions.

## 2 Methods

### 2.1 Study population

We retrospectively collected clinical data from patients presenting to the emergency department with an acute stroke between May 2019 and March 2023. Inclusion criteria: (1) Stroke definition and diagnosis consistent with the 2013 American Heart Association/American Stroke Association (AHA/ASA) updated definitions (9), (2) Within 24 h of onset; (3) Age >18 years; (4) First-ever stroke; (5) Glasgow Coma Scale (GCS) score >8; (6) Signed informed consent. Exclusion criteria: (1) Concomitant coagulopathy; (2) Pre-existing extracranial thrombotic or hemorrhagic complications; (3) Hepatic insufficiency; (4) Malignancy; (5) Long-term anticoagulant therapy; (6) Subarachnoid hemorrhage or intraventricular hemorrhage. Patients with GCS ≤ 8 were excluded to reduce confounding from complex intensive care interventions (such as mechanical ventilation, decompressive craniectomy, and advanced hemodynamic management) on coagulation factor activity. Patients received standard care. Hemorrhagic stroke (HS) was treated according to the 2015 AHA/ASA Guidelines for the Management of Spontaneous Intracerebral Hemorrhage (10). Ischemic stroke (IS) was treated according to the 2018 AHA/ASA Early Management of Patients With Acute Ischemic Stroke guidelines (11). The research was carried out according to the Helsinki Declaration and was approved by the Ethics Committee of Ruijin Hospital affiliated with Shanghai Jiao Tong University School of Medicine. All patients or family members signed a written informed consent form.

### 2.2 Baseline data

Acquisition All patients were evaluated by two trained emergency physicians. Demographics, vital signs, and baseline clinical data were recorded, including body mass index, GCS score, and National Institutes of Health Stroke Scale (NIHSS) score. Participants provided history, including prior anticoagulation therapy, smoking status, hypertension (HTN), diabetes (DM), hyperlipidemia (HL), and coronary artery disease (CAD). Routine blood tests, including blood count, liver and renal function, coagulation function, fasting blood glucose, and lipids, were completed within 24 h of admission. Cranial CT or MRI imaging was completed within 24 h of admission. Lesion volumes on computed tomography (CT) and diffusion-weighted imaging (DWI) were evaluated using MIPAV software (version 11.0) by two experienced radiologists, who were blinded to the clinical and laboratory findings.

### 2.3 Specimen test

Citrated blood samples were obtained within 24 h of admission and delivered to the hospital’s central laboratory for the measurement of coagulation factor activity levels, and blood collection was completed before any major intervention that could affect coagulation activity, such as blood transfusion, reversal agents, thrombolysis, or surgery. These were measured using one-stage coagulation assays and reported as a percentage of normal pooled plasma activity (standard activity units). The testers were blinded to participant characteristics.

### 2.4 Outcomes

The primary endpoint was the neurological outcome at 90 days post-onset. A trained physician who was unaware of the coagulation factor data conducted standardized and structured telephone follow-up interviews with the patients themselves or their family members 90 days after the onset of the disease to obtain the Modified Rankin Scale (mRS) score. The telephone follow-up evaluators were blinded to the clinical records and the entire diagnostic and treatment process. A favorable prognosis was defined as an mRS score ≤ 2.

### 2.5 Grouping

Patients were divided by stroke type into hemorrhagic stroke and ischemic stroke. According to the Modified Rankin Scale, patients were divided into a favorable prognosis group (mRS ≤ 2) and an unfavorable prognosis group (mRS > 2).

### 2.6 Statistical analysis

SPSS 23.0 and MedCalc were used for statistical analysis. Categorical variables were expressed as frequencies (composition ratios) and compared using the $\chi^{2}$ test. Non-normally distributed continuous variables were expressed as medians (interquartile ranges) and compared using the Mann–Whitney U test or Kruskal-Wallis test. Spearman’s rank correlation was calculated for different variables. Multivariable logistic regression models were developed to evaluate the association between coagulation factor activity and 90-day neurological outcomes. We employed a complete-case analysis (listwise deletion) to manage missing data. Of the 181 patients included in the initial baseline analysis, 10 patients (6 with hemorrhagic stroke and 4 with ischemic stroke; 5.5% of the total cohort) were excluded from the multivariable regression analyses due to incomplete coagulation factor data. Consequently, the final regression models were based on 171 patients. In order to avoid collinearity and model instability, multivariable regression models were constructed separately for each of the four coagulation factors (FVII, FIX, FXI, FXII). Model 1 was adjusted for age and sex, the events per variable (EPV) was 14.3; Model 2 was further adjusted for smoking history, hypertension history, admission blood glucose, and NIHSS score, the EPV was 6.1. Results were expressed as odds ratios (OR) and their 95% confidence intervals (CI). All tests were two-sided, and *p* < 0.05 was considered statistically significant. The lower limit of CRP was 10 mg/L. For the value below the lower limit of detection, the value was assigned to half of the lower limit of detection (i.e., 5 mg/L) for the sake of quantitative analysis. GraphPad Prism 8.0.2 was used for plotting.

## 3 Results

### 3.1 Study population characteristics

**Figure 1:**
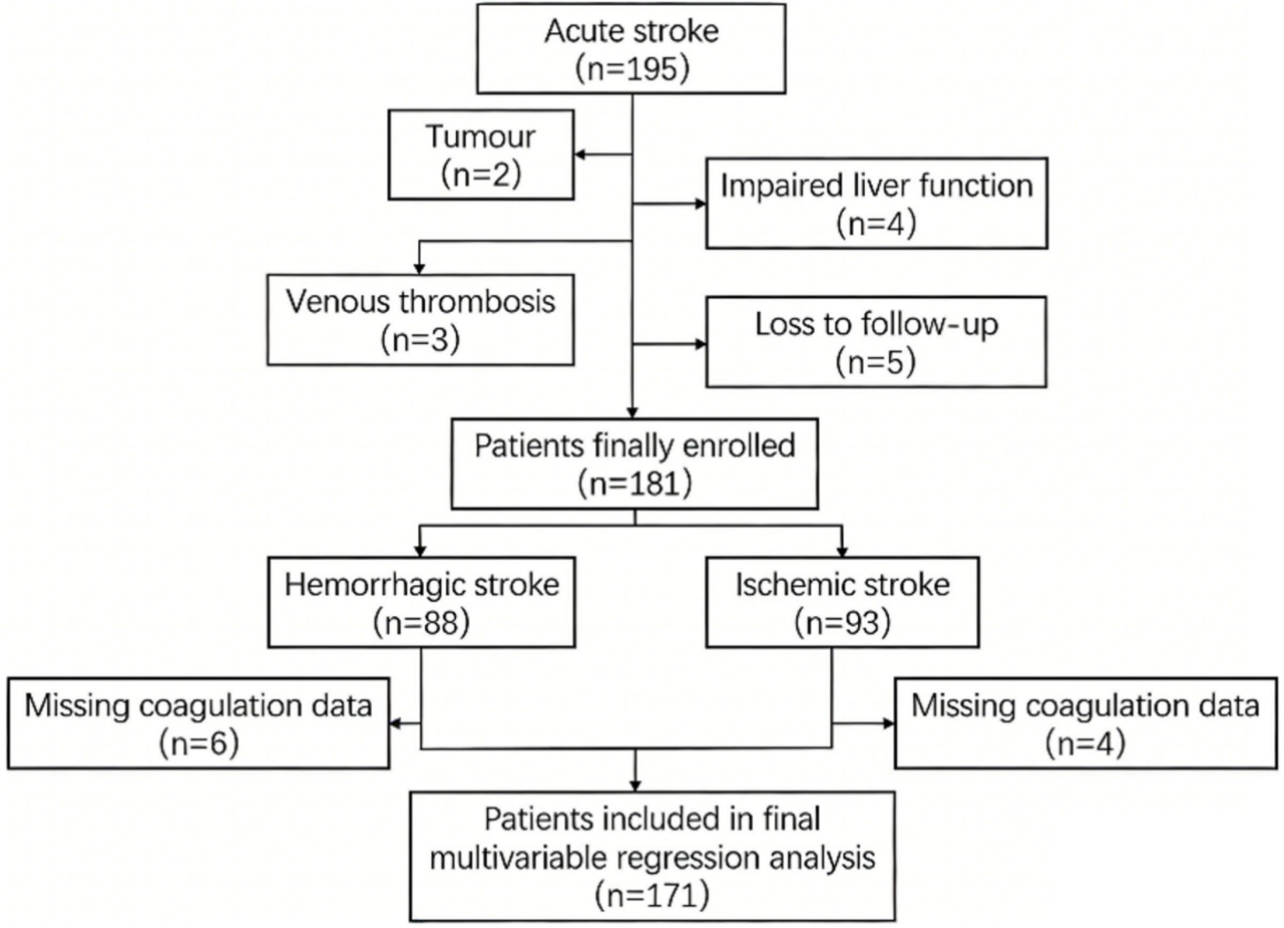
Flow diagram of patient enrollment and study population analysis.

A total of 195 patients were enrolled in this study. After excluding 14 patients due to malignancy (*n* = 2), hepatic insufficiency (*n* = 4), venous thrombosis (*n* = 3), and loss to follow-up (*n* = 5), 181 patients were included in the baseline analysis (Table 1). For the final multivariable regression models, 10 additional patients were excluded due to incomplete coagulation factor data, resulting in a final analytical sample of 171 patients (Figure 1). Sensitivity analyses comparing baseline characteristics between included and excluded patients revealed no systematic differences (Supplementary Table 1).

**Table 1:** Baseline characteristics of all participants at admission.

Baseline characteristic	ALL (*N* = 181)	IS (*N* = 93)	HS (*N* = 88)	*p*
Age, y (IQR)	64 (54,73)	67 (57,76)	58 (50,71)	0.001
Male, *n* (%)	120 (64.1)	55 (59.1)	61 (69.3)	0.154
NIHSS, median (IQR)	10 (6–15)	9 (6–12)	13 (8–17)	0.001
GCS, median (IQR)	14 (12–15)	15 (13–15)	13 (11–15)	<0.001
MAP, mmHg (IQR)	93 (86–106)	92 (85–105)	94 (87–106)	0.524
Hypertension, *n* (%)	130 (71.8)	68 (73.1)	62 (70.5)	0.691
Diabetes mellitus, *n* (%)	50 (27.6)	28 (30.1)	22 (25.0)	0.442
Hypercholesterolemia, *n* (%)	55 (30.4)	25 (26.9)	30 (34.1)	0.292
Smoking, *n* (%)	51 (28.2)	26 (28.0)	25 (28.4)	0.752
Lesion volume (cm^3^)	5.6 (2.0,14.1)	4.8 (1.7,10.6)	8.3 (3.1,23.5)	0.088
White blood cells, ×10^9^/L (IQR)	7.84 (6.47,9.64)	7.36 (6.38,9.01)	8.50 (6.57,10.95)	0.114
Glucose, mmol/L (IQR)	7.3 (6.0,9.4)	7.2 (6.0,10.7)	7.4 (5.9,8.8)	0.614
Platelet (×10^9/^L) (IQR)	180 (158,223)	182 (161,235)	179 (151,220)	0.867
HbA1c,%(IQR)	5.9 (5.6,6.4)	5.9 (5.6,7.3)	5.8 (5.4,6.2)	0.325
Total cholesterol, mmol/L (IQR)	4.6 (3.9,5.5)	4.6 (4.2,5.2)	4.6 (3.7,6.0)	0.712
Creatinine, μmoI/L (IQR)	77 (66,86)	77 (67,83)	78 (62,97)	0.964
INR (IQR)	1.02 (0.97,1.08)	1.01 (0.96,1.06)	1.04 (1.00,1.10)	0.023
CRP, mg/L (IQR)	5 (5,5)	5 (5,5)	5 (5,13)	0.431
Ca, mmol/L (IQR)	2.28 (2.22,2.36)	2.30 (2.24,2.35)	2.25 (2.17,2.36)	0.114

Continuous variables are presented as median (interquartile range, IQR), and categorical variables as frequency (percentage). *p* values represent comparisons between the ischemic stroke group and the hemorrhagic stroke group. IS, ischemic stroke; HS, hemorrhagic stroke; GCS, Glasgow Coma Scale; NIHSS, National Institutes of Health Stroke Scale; MAP, Mean Arterial Pressure; HbA1c, Hemoglobin A1c; INR, International Normalized Ratio; CRP, C-reactive protein.

The 181 patients included in baseline evaluation had a median age of 64 years (IQR 54–73), and 120 (64.1%) were male. Clinical outcomes at 90 days showed that 88 (48.6%) patients experienced an unfavorable prognosis (mRS > 2). Hypertension was the most prevalent cardiovascular risk factor.

### 3.2 Baseline characterization

Baseline characteristics of participants at admission are shown in Table 1. Compared with ischemic stroke, patients with hemorrhagic stroke were younger (IQR 58 vs. 67 years, *p* = 0.001), had higher stroke severity (median NIHSS 13 vs. 9, *p* = 0.001), more severe disturbance of consciousness (median GCS 13 vs. 15, *p* < 0.001), and higher INR levels (median 1.04 vs. 1.01, *p* = 0.023). There were no significant differences between the two groups in gender composition, cardiovascular risk factors, Lesion volume, blood routine, or biochemical indicators.

### 3.3 Coagulation factor activity and stroke prognosis

**Figure 2:**
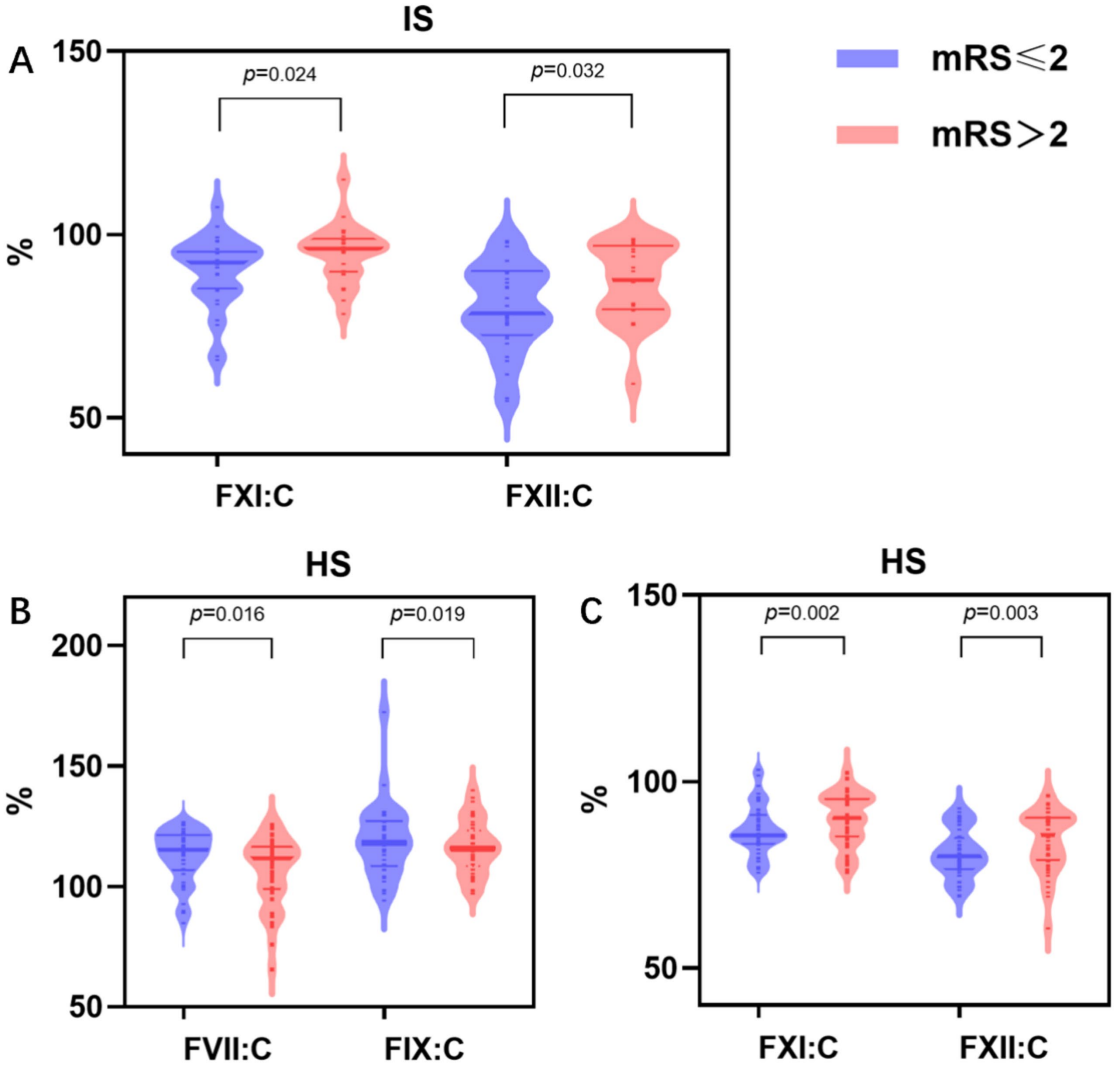
Correlation analysis between coagulation factor activity and stroke prognosis. **(A)** Correlation between FXI:C, FXII:C and unfavorable prognosis of IS; **(B)** Correlation between FVII:C, FIX:C and unfavorable prognosis of HS; **(C)** Correlation between FXI:C, FXII:C and unfavorable prognosis of HS. HS, hemorrhagic stroke; IS, ischemic stroke; FVII:C, coagulation factor VII activity; FIX:C, coagulation factor IX activity; FXI:C, coagulation factor XI activity; FXII:C, coagulation factor XII activity.

*Ischemic stroke*: Among 93 IS patients, compared with the favorable prognosis group (*n* = 48), the unfavorable prognosis group (*n* = 45) had significantly higher FXI activity (median 96.1% vs. 92.4%, p=0.024 ) and FXII activity (median 87.6% vs. 78.5%, *p* = 0.023) (Figure 2A). FVII and FIX activities showed no significant differences between groups.

*Hemorrhagic stroke*: Among 88 HS patients, compared with the favorable prognosis group (mRS ≤ 2, *n* = 45), the unfavorable prognosis group (mRS > 2, *n* = 43) had significantly lower FVII activity (median 111.7% vs. 115.3%, P=0.016 ) and FIX activity (median 115.6% vs. 118.1%, p=0.019 ) (Figure 2B), while FXI activity was significantly higher (median 90.3% vs. 85.6%, *p* = 0.002 ) and FXII activity was significantly higher (median 85.7% vs. 80.0%, *p* = 0.003) (Figure 2C).

### 3.4 Multivariable logistic regression analysis

Based on known important baseline variables and the results of univariate analyses (Supplementary Table 2), we performed multivariable logistic regression analysis for coagulation factors (FVII, FIX, FXI, FXII) related to prognosis in patients with hemorrhagic stroke (Table 2).

**Table 2:** Analysis of coagulation factors in multivariate logistic regression for 90-day unfavorable prognosis in patients with hemorrhagic stroke.

Variables	Model 1 OR (95% CI)	*p*	Model 2 OR (95% CI)	*p*
FVII:C (per 10% increase)	0.603 (0.398–0.914)	0.017	0.464 (0.250–0.863)	0.015
FIX:C (per 10% increase)	0.535 (0.297–0.966)	0.038	0.498 (0.249–0.998)	0.049
FXI:C (per 10% increase)	2.838 (1.287–6.256)	0.010	1.739 (0.629–4.806)	0.286
FXII:C (per 10% increase)	2.840 (1.327–6.080)	0.007	2.725 (1.098–6.762)	0.031

Model 1: Adjusted for age and sex. Model 2: Adjusted for age, sex, smoking status, hypertension, blood glucose, and NIHSS score. All OR values were calculated for each 10% increase in coagulation factor activity. FVII:C, coagulation factor VII activity; FIX:C, coagulation factor IX activity; FXI:C, coagulation factor XI activity; FXII:C, coagulation factor XII activity.

In Model 1, adjusted only for age and sex, FVII, FIX, FXI, and FXII activities were significantly associated with prognosis in hemorrhagic stroke (all *p* < 0.05). Given the limited number of outcome events and to assess robustness against overfitting, we further evaluated these associations using Model 1 as a parsimonious sensitivity framework. In these simplified models, the addition of each coagulation factor to the base model (age and sex) yielded modest improvements in discrimination, with AUCs ranging from 0.720 to 0.758 (Supplementary Table 3), and all four factors remained independently associated with prognosis (*p* < 0.05).

In Model 2, which additionally adjusted for smoking, hypertension, blood glucose, and NIHSS score, FVII activity (per 10% increase, OR = 0.464, 95% CI: 0.250–0.863, *p* = 0.015) and FIX activity (per 10% increase, OR = 0.498, 95% CI: 0.249–0.998, *p* = 0.049) remained independently negatively associated with an unfavorable prognosis; FXII activity (per 10% increase, OR = 2.725, 95% CI: 1.098–6.762, *p* = 0.031) remained independently positively associated with an unfavorable prognosis. FXI activity lost statistical significance in the fully adjusted model (*p* = 0.286), likely reflecting its partial correlation with stroke severity.

In the IS group, FXI and FXII activities showed no statistical significance after adjustment in Model 2.

### 3.5 Correlation analysis

**Figure 3:**
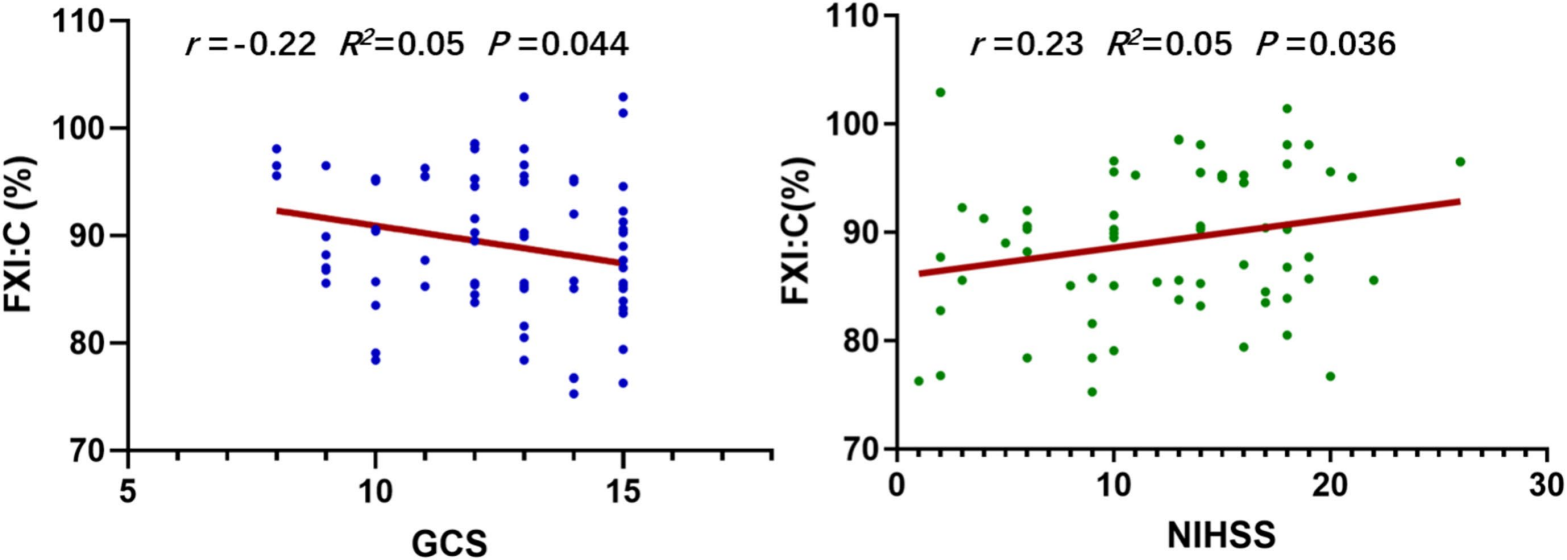
Coagulation factors and prognostic outcomes in patients with cerebral hemorrhage. FXI:C, coagulation factor XI activity; GCS, Glasgow Coma Scale; NIHSS, National Institutes of Health Stroke Scale.

In hemorrhagic stroke patients, correlation analysis showed that FXI activity was mildly negatively correlated with GCS score (r = −0.22, P=0.044 ) and mildly positively correlated with NIHSS score (r = 0.23, p=0.036 ) (Figure 3), suggesting that FXI activity may slightly associated with stroke severity. FVII, FIX, and FXII activities showed no significant correlation with GCS, NIHSS, or lesion volume (*p* > 0.05).

## 4 Discussion

This study provides an analysis of the prognostic value of coagulation factors VII, IX, XI, and XII in the acute phase of stroke. Our findings reveal a type-specific prognostic profile: FVII and FIX activities appear to serve as protective markers in hemorrhagic stroke, where reduced levels are associated with worse 90-day functional recovery. In contrast, elevated FXII activity emerges as an independent associated factors of unfavorable outcomes across stroke subtypes. Notably, although FXI activity was significantly associated with stroke prognosis in univariate analysis, this association was attenuated after adjusting for established disease severity markers, particularly the NIHSS score, suggesting that FXI may function as a surrogate biomarker for stroke severity rather than an independent pathogenic mediator.

The protective roles of FVII and FIX in hemorrhagic stroke are particularly noteworthy. Previous research has explored exogenous rFVIIa as a therapeutic agent to stabilize the microvascular endothelium via the TF–FVIIa–FXa pathway, aiming to mitigate progressive hemorrhage (3). However, the therapeutic utility of rFVIIa remains controversial, as clinical trials have yielded inconsistent results regarding functional recovery and mortality (12, 13). Our findings suggest that endogenous levels of FVII and FIX are distinct from the pharmacodynamic effects of rFVIIa infusion. Given that FVII activity was not correlated with initial stroke severity (NIHSS/GCS) but remained an independent predictor, it is plausible that endogenous FVII and FIX reflect the patient’s intrinsic hemostatic reserve (14, 15), which is essential for maintaining microvascular integrity and limiting initial hematoma expansion. Thus, these factors might offer a baseline assessment of a patient’s capacity to tolerate hemorrhagic insult. However, the potential for overfitting cautions against over-interpreting these findings, and the explanatory power of these associations should be considered provisional.

The close association between FXII activity and adverse outcomes in stroke subtypes may be attributable to its potential role in the interplay between coagulation and inflammation (16, 17). FXII functions as a bidirectional regulator; beyond its role in intrinsic coagulation, its activation product, FXIIa, initiates the kallikrein–kinin cascade, thereby amplifying inflammatory responses and aggravating cerebral edema (3, 18, 19). While FXII deficiency has been associated with reduced infarct volume in ischemic stroke models (18, 19), our data extend this to hemorrhagic stroke, postulating that FXII-driven inflammatory pathways may exacerbate post-stroke neural injury. The divergence between our findings and previous long-term cohort studies (8, 20) regarding secondary event incidence may be attributed to differences in clinical endpoints—namely, our focus on acute-phase neurological recovery versus long-term thrombotic recurrence. This suggests that FXII may primarily modulate the acute inflammatory sequelae of stroke rather than the chronic risk of vascular events.

Regarding FXI, the loss of independent prognostic significance after multivariable adjustment, coupled with its slightly correlation with NIHSS and GCS scores, indicates that FXI activity is likely an acute-phase reactant mirroring stroke severity rather than an independent driver of neurological decline. These results contrast with large-scale cardiovascular studies suggesting FXI as a potential therapeutic target (21, 22). This discrepancy may reflect temporal dynamics; FXI levels may fluctuate relative to the severity of the inflammatory insult during the acute stroke phase, thereby acting as a barometer of initial injury rather than an endogenous causal risk factor.

### 4.1 Limitations

Several limitations should be addressed. First, the single-center, retrospective design potentially limits the external validity and generalizability of our findings. Second, to avoid the influence of complex treatments in critically ill patients on the coagulation system, this study excluded patients with severe neurological deficits (GCS ≤ 8), which may introduce selection bias, and the study cohort cannot fully represent severe stroke patients. Third, although 10 patients (5.5%) were excluded due to missing coagulation-related data, no statistically significant baseline differences were detected between the included and excluded cohorts, though the small sample size limited the inference. Finally, coagulation measurements at admission provide only a cross-sectional snapshot. We did not include lesion volume or GCS scores in the final adjusted models, and this decision may introduce the risk of residual confounding. Further multicenter, longitudinal studies are warranted to validate these findings and elucidate the dynamic changes in coagulation activity during the subacute phase of stroke recovery.

## 5 Conclusion

Decreased FVII and FIX activities were independent predictors of poor prognosis in hemorrhagic stroke, while elevated FXII activity was associated with poor outcomes. Early coagulation factor activities showed subtype-specific associations with 90-day functional outcomes, particularly in hemorrhagic stroke; however, these exploratory findings require external validation before clinical implementation.

## Data Availability

The original contributions presented in the study are included in the article/Supplementary material, further inquiries can be directed to the corresponding authors.
