# Supplementary material for: Prognostic significance of coagulation factor activity in acute stroke: a retrospective cohort study
Source: Front Med (Lausanne). 2026 May 29;13:1860924. doi: 10.3389/fmed.2026.1860924 (PMC13260378; doi:10.3389/fmed.2026.1860924)
Supplement: Supplementary file 1 [file Supplementary_Table_1.PDF]

Supplementary Table S1 Baseline characteristics of the included cohort (n=171) versus the excluded cohort (n=10) due to missing coagulation data.

Characteristics	Included (*n*=171)	Excluded (*n*=10)	*P*-value
Age, y	64 (54–73)	66 (54–74)	0.762
Male, n (%)	113 (66.1%)	7 (70.0%)	0.799
NIHSS score	10 (6–15)	11 (7–16)	0.068
GCS score	14 (12–15)	14 (13–15)	0.991
Hypertension, n (%)	123 (71.9%)	7 (70.0%)	0.895
Diabetes mellitus, n (%)	48 (28.1%)	2 (20.0%)	0.579
Hypercholesterolemia, n (%)	52 (30.4%)	3 (30.0%)	0.978
Smoking, n (%)	49 (28.7%)	2 (20.0%)	0.554

Note: Continuous variables are presented as median (interquartile range, IQR), and categorical variables as frequency (percentage). *P*-values were calculated using the Mann-Whitney U test.
