# Supplementary material for: Prognostic significance of coagulation factor activity in acute stroke: a retrospective cohort study
Source: Front Med (Lausanne). 2026 May 29;13:1860924. doi: 10.3389/fmed.2026.1860924 (PMC13260378; doi:10.3389/fmed.2026.1860924)
Supplement: Supplementary file 2 [file Supplementary_Table_2.PDF]

Supplementary Table S2. Univariate regression analysis for 90-day unfavorable prognosis in hemorrhagic stroke patients

	mRS≤2 (n=45)	mRS＞2 (n=43)	*P*
Age,y (IQR)	53(45, 62)	63(56, 71)	0.016
Male,n(%)	31(68.9)	30(69.8)	0.929
Smoking,n(%)	8(17.8)	17(39.5)	0.024
Hypertension,n(%)	28(62.2)	34(79.1)	0.083
Glucose,mmol/L (IQR)	6.4(5.7, 7.5)	8.2(7.8, 9.2)	＜0.001
NIHSS,median (IQR)	9(4, 13)	15(13, 18)	＜0.001
FVII:C(%)	113.9(109.7, 118.8)	112.3(99.4, 116.6)	0.016
FIX:C(%)	123.8(114.4, 129.9)	115.6(109.5, 120.3)	0.019
FXI:C(%)	85.6(84.5, 91.7)	92.6(85.6, 95.6)	0.002
FXII:C(%)	80.0(77.4, 83.2)	87.3(79.1, 90.1)	0.003

Abbreviations: FVII:C, coagulation factor VII activity; FIX:C, coagulation factor IX activity; FXI:C, coagulation factor XI activity; FXII:C, coagulation factor XII activity.
