# Supplementary material for: Prognostic significance of coagulation factor activity in acute stroke: a retrospective cohort study
Source: Front Med (Lausanne). 2026 May 29;13:1860924. doi: 10.3389/fmed.2026.1860924 (PMC13260378; doi:10.3389/fmed.2026.1860924)
Supplement: Supplementary file 3 [file Supplementary_Table_3.PDF]

Supplementary Table 3 Area under the ROC Curve (AUC) for Predicting Poor Prognosis of Stroke by Different Models

	AUC (95% CI)	ΔAUC
**Model 1**	0.675(0.555-0.794)	-
**Model 1 + FVII:C**	0.720(0.609-0.830)	+0.045
**Model 1 + FIX:C**	0.758(0.611-0.904)	+0.083
**Model 1 + FXI:C**	0.736(0.621-0.852)	+0.061
**Model 1 + FXII:C**	0.746(0.640-0.852)	+0.071

Note: Model 1: age and sex; AUC, area under the receiver operating characteristic curve; CI, confidence interval; ΔAUC, increase in AUC compared with the model 1; FVII:C, coagulation factor VII activity; FIX:C, coagulation factor IX activity; FXI:C, coagulation factor XI activity; FXII:C, coagulation factor XII activity.
